# Towards Generalizable Deepfake Detection: An Inconsistency-Aware Vision–Language Model Tuning Approach

**DOI:** 10.3390/s26144512

**Published:** 2026-07-16

**Authors:** Lu Zhang, Shufan Peng, Mingle Xu, Tianliang Lu

**Affiliations:** 1 College of Information and Cyber Security, People’s Public Security University of China, Beijing 100038, China; gadxyjsyzl@163.com (L.Z.);; 2 Department of Investigation, Shandong Police College, Jinan 250200, China; 3 School of Computer Science and Information Security, Guilin University of Electronic Technology, Guilin 541004, China; xml@jbnu.ac.kr; 4 Laboratory for Digital and Intelligent Prevention and Control of Societal Security Risks, People’s Public Security University of China, Beijing 100038, China

**Keywords:** inconsistency, vision–language models, IncoTune, deepfake detection

## Abstract

Deepfakes generated by advanced AI models pose growing challenges to digital media authenticity. Large Vision–Language Models (VLMs) have recently been explored for image forensics due to their multimodal representation ability. However, many existing VLM-based deepfake detection methods keep the visual encoder fixed to preserve pre-trained knowledge, which may limit the model’s sensitivity to low-level inconsistency artifacts that are important for deepfake detection. To address this issue, we propose IncoTune, an inconsistency-aware tuning framework that introduces trainable vision-side adaptation into the visual encoder and applies Directional Low-Rank Adaptation (DoRA) to selected linear projection layers in both the visual encoder and the language model. We further report an empirical observation in the ablation study: under the evaluated adapter configuration, replacing LoRA with DoRA in the frozen-vision setting does not improve the average AUC, whereas DoRA provides additional gains when combined with vision-side adaptation. Experimental results on FaceForensics++, DFD, Celeb-DF, DFDC, and DFDCP show that IncoTune improves cross-dataset frame-level detection performance over the frozen-vision baseline and achieves competitive performance among representative frame-level methods, while updating only 27.0M adapter parameters during training. Robustness experiments further evaluate the model behavior under common image degradations.

## 1. Introduction

Recent advances in deep learning, particularly Generative Adversarial Networks (GANs) and Diffusion Models, have enabled the generation of highly realistic synthesized media, commonly known as deepfakes. While these technologies have useful applications in digital entertainment and creative industries, they can also be misused for disinformation, identity fraud, and political manipulation. Therefore, developing reliable deepfake detection systems has become an important research topic in computer vision [1,2].

Historically, deepfake detection has been dominated by Convolutional Neural Networks (CNNs) explicitly trained to capture specific manipulation traces, such as blending boundaries, up-sampling artifacts, or frequency-domain anomalies. While these conventional methods often achieve strong performance on their respective training datasets, they may suffer from substantial performance degradation when exposed to unseen generative models or novel manipulation techniques. This cross-dataset generalization problem is often related to the tendency of task-specific CNNs to overfit to dataset-specific biases rather than learning generalizable forgery cues.

Recently, Large Vision–Language Models (VLMs), such as LLaVA, InternVL, and Qwen-VL [3], have shown strong performance in multimodal understanding. Based on their pre-trained visual-language representations, researchers have begun exploring VLMs for image forensics via prompt engineering and parameter-efficient fine-tuning (PEFT). Many existing VLM-based forensic frameworks adopt a frozen-vision paradigm, where the visual encoder is kept fixed and parameter updates are mainly applied to the language model or lightweight adaptation modules.

We argue that strictly freezing the visual encoder is not always suitable for deepfake detection tasks. Pre-trained visual encoders are mainly optimized to capture high-level semantic invariance, which is useful for general visual understanding but may be less sensitive to subtle local variations. In deepfake detection, such variations may contain important forgery cues, including pixel-level discrepancies, texture anomalies, and blending inconsistencies. Therefore, a fixed visual encoder may provide limited task-adaptive artifact-related information to the downstream language model.

**Figure 1:**
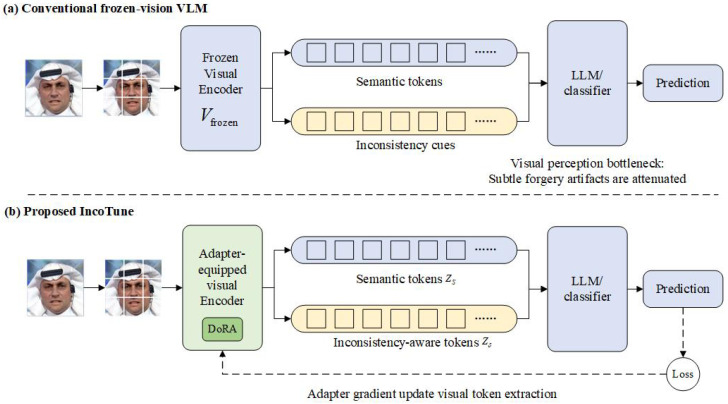
Conceptual comparison between the conventional frozen-vision paradigm and the proposed vision-side adaptation framework.

As conceptually illustrated in Figure 1, to address this bottleneck, we propose IncoTune, an inconsistency-aware tuning approach. Different from common VLM-based PEFT settings that mainly adapt the language side while keeping the visual pathway fixed, IncoTune introduces trainable adapter updates into the visual encoder. The original pre-trained weights are kept frozen, while Directional Low-Rank Adaptation (DoRA) modules are applied to selected linear projection layers in both the visual encoder and the language model. This design allows the visual token extraction process to be adapted to the deepfake detection task without full-parameter fine-tuning.

Our ablation study further reports an empirical observation about the interaction between adapter type and adapter placement. Under the frozen-vision setting, replacing LoRA with DoRA does not improve the average AUC in our experiments. When trainable adaptation is introduced into the visual encoder, DoRA provides additional gains over the corresponding LoRA variant. This observation suggests that the effect of DoRA depends on whether the visual representation is also adapted to task-specific forgery cues, rather than being determined by the adapter type alone.

Our main contributions are summarized as follows:

- We investigate the frozen-vision setting in VLM-based deepfake detection and analyze its potential limitation for frame-level deepfake detection. Instead of treating the visual encoder as a fixed feature extractor, we examine whether introducing trainable adaptation into the visual pathway can improve cross-dataset generalization.
- We propose IncoTune, a vision-side parameter-efficient fine-tuning (vision-side PEFT) framework for deepfake detection. The original pre-trained weights are kept frozen, while DoRA modules are inserted into linear projection layers in both the visual encoder and the language model. This design allows the visual token extraction process to be adapted to forgery cues without full-parameter fine-tuning.
- We report an empirical observation regarding the interaction between adapter type and vision-side adaptation. In our ablation setting, applying DoRA only under a frozen visual encoder does not improve the average AUC, whereas combining DoRA with vision-side adaptation provides additional gains over the corresponding LoRA variant. This result indicates that adapter placement should be considered together with adapter type in VLM-based deepfake detection.
- Frame-level experiments on FF++-to-unseen cross-dataset settings show that IncoTune improves over the frozen-vision ablation baseline and achieves competitive performance among representative frame-level detectors. Robustness experiments further evaluate the model under common image degradations.

## 2. Related Work

### 2.1. Artifact Mining and Inconsistency Detection

Many deepfake detection methods aim to expose subtle structural discrepancies, blending artifacts, or frequency anomalies inadvertently introduced during the synthetic generation process. Early methods often used Convolutional Neural Networks (CNNs) to capture pixel-level artifacts. For instance, Face X-ray [4] identified blending boundaries characteristic of face-swapping by explicitly locating spatial inconsistencies between the manipulated inner face and the unaltered background. Building upon this, Self-Blended Images (SBI) [5] proposed a training strategy that artificially synthesizes blending artifacts to encourage the network to learn generalized forgery traces.

As generative models became more capable of producing visually realistic face manipulations, researchers increasingly explored frequency-domain cues. F3-Net [6] introduced frequency-aware spatial–temporal networks to mine frequency-level anomalies caused by up-sampling, while SPSL [7] utilized dual-branch designs to simultaneously capture spatial and phase shallow learning features. Furthermore, SRM [8] showed the effectiveness of using high-frequency noise features to uncover subtle generative traces.

More recently, researchers have explored more general forgery cues beyond manually defined artifacts. Frameworks such as CFM [9] and ED [10] have been proposed to mine manipulation clues that traditional detectors may overlook. Concurrently, micro-level texture and patch inconsistencies have gained attention. Methods like PatchCraft [11] use patch reconstruction to reduce the reliance on holistic facial semantics and encourage the network to focus on inter-pixel correlation anomalies. Similarly, reconstruction–classification learning (e.g., RECCE [12]) reconstructs pristine faces to highlight the structural anomalies of forged inputs. Although these methods perform well on specific distributions, their task-specific architectures may limit their ability to adapt to unseen synthesis methods.

### 2.2. Cross-Dataset Generalization in Deepfake Detection

Cross-dataset generalization remains a major challenge in image and video forensics. Detectors may overfit to the fingerprints of specific generative models, leading to performance degradation on unseen domains. To address this, many studies have explored robust, domain-agnostic representation learning.

One prominent approach involves data augmentation and feature-space simulation. For example, Latent Space Augmentation (LSA) [13] aims to reduce forgery-specific overfitting by simulating potential and diverse generative artifacts directly within the latent feature space. Another line of work mitigates identity and semantic biases. Recent advancements like SELFI [14] employ selective fusion of identity features, while UDD [15] explores unbiased deepfake detection via token-level shuffling and mixing, both aiming to prevent the model from overfitting to specific facial identities. Contrastive learning has also been widely adopted to construct generalized boundaries. DCL [16] utilizes dual contrastive learning to separate pristine and forged representations, and UCF [17] explicitly uncovers common forgery features across disparate manipulation types.

In the video domain, researchers have exploited temporal coherence to achieve generalizability. Methods such as NACO [18] learn natural consistency representations, and TALL++ [19] designs specialized thumbnail layouts to capture spatial–temporal anomalies.

Recent studies have increasingly explored pre-trained foundation models for generalizable deepfake detection. UnivFD [20] showed that applying a linear probe over the frozen visual encoder of CLIP can provide strong generalization ability. Similarly, recent approaches such as GenD [21] use vision transformers to improve cross-benchmark detection. However, learning a feature space that generalizes well without severe forgetting remains challenging.

### 2.3. Adapting Vision–Language Models for Deepfake Detection

Large Vision–Language Models (VLMs), such as LLaVA, InternVL, and Qwen-VL, have shown strong multimodal reasoning and zero-shot perception abilities. These models have therefore attracted increasing attention in image forensics. Early explorations formulated deepfake detection as a Visual Question Answering (VQA) task. AntifakePrompt [22] optimized lightweight soft prompts to guide a frozen InstructBLIP model toward artifact identification. To further bridge the gap between general multimodal understanding and specialized forensics, researchers have proposed model reprogramming techniques (e.g., RepDFD [23]), mapping forensic-specific visual features into the frozen VLM semantic space.

As parameter-efficient fine-tuning (PEFT) techniques have matured, recent methods have increasingly adopted lightweight adapters. For instance, ForAda [24] explicitly adapts the CLIP architecture for generalizable face forgery detection, and FCG [25] employs facial component-guided adaptation to enhance the foundation model’s sensitivity to localized artifacts. Concurrently, comprehensive benchmarks like DFBench [26] evaluated the deepfake detection capability of state-of-the-art open-source VLMs, utilizing Low-Rank Adaptation (LoRA) as a standard baseline.

Recent VLM-based deepfake detection methods often keep the visual encoder fixed to preserve pre-trained visual-language knowledge, while adapting the language model or lightweight modules for the forensic task. Recent studies have begun to recognize the limitations of strictly frozen visual representations. For instance, Yermakov et al. [27] explored strategies to improve the use of CLIP representations for generalizable deepfake detection. Similarly, to address temporal dynamics in manipulated videos, concurrent works such as Tong et al. [28] proposed adapting later vision layers together with spatiotemporal modeling. However, these methods either focus on contrastive vision–language models or rely on temporal modeling. In the context of generative VLMs tuned via low-rank adaptation, the interaction between vision-side adapter updates and adapter type has received less attention.

In this paper, we revisit the assumption that the visual encoder should always remain fixed in VLM-based forensics. A fixed visual encoder can preserve useful pre-trained representations, but it may also limit task-specific adaptation to subtle local artifacts. Since deepfake traces often appear as local texture, boundary, or pixel-level inconsistencies, the frozen-vision setting may restrict the downstream language model’s access to fine-grained forgery cues. This motivates the proposed vision-side parameter-efficient adaptation framework.

## 3. Materials and Methods

### 3.1. Problem Formulation

Let $X\in R^{H\times W\times C}$ denote an input image, which belongs to either the pristine domain $D_{\mathrm{real}\;}$ or the forged domain $D_{\mathrm{fake}}$. A generated or manipulated image $X_{\mathrm{fake}\;}$ can be abstracted as the synthesis of pristine semantics and generative noise:

(1) $$X_{\mathrm{fake}}=\left(1-M\right)⊙X_{\mathrm{real}}+M⊙G\left(z_{g}\right)+\delta .$$

where *M* is a spatial mask denoting the manipulated region, $G(z_{g})$ represents the synthesized content generated from a generative latent variable, and δ denotes subtle inconsistency-related artifacts that may be introduced during generation or manipulation, such as up-sampling noise, blending boundary artifacts, or spectral anomalies.

The objective of VLM-based deepfake detection is to learn a mapping function $F_{\theta }:X\to y$, where y∈{0,1} denotes the binary authenticity label. The prediction is conditioned on a textual prompt *T*, and the probability of forgery is formulated as

(2) $$P\left(y=1|X,T\right)=F_{\theta }\left(X,T\right).$$

Let $\theta =\{\theta_{0},\theta_{a}\}$, where $\theta_{0}$ denotes the frozen pre-trained parameters and $\theta_{a}$ denotes the trainable adapter parameters. Our goal is to optimize $\theta_{a}$ to distinguish authentic and forged inputs across unseen domains.

### 3.2. The Visual Perception Bottleneck

A standard VLM comprises a visual encoder V (typically a Vision Transformer), a cross-modal projector P, and a Large Language Model (LLM) L. Conventional parameter-efficient fine-tuning (PEFT) strategies often freeze the visual encoder V to reduce computational cost and preserve pre-trained knowledge. Consequently, the visual representation is extracted as $Z=V_{\mathrm{frozen}}\left(X\right)$.

However, $V_{\mathrm{frozen}}$ is usually pre-trained on large-scale natural image-text pairs, where the learning objective encourages the model to capture high-level semantic invariance. Such semantic abstraction is useful for general visual understanding, but it may reduce the sensitivity of the representation to subtle local variations. In the context of deepfake detection, these local variations may include pixel-level discrepancies, texture anomalies, blending boundaries, and other inconsistency-related cues.

In this setting, a real image and its visually similar fake counterpart may obtain close representations in the semantic feature space S, especially when the frozen encoder mainly preserves high-level semantic information:

(3) $${∥V_{\mathrm{frozen}}\left(X_{\mathrm{real}}\right)-V_{\mathrm{frozen}}\left(X_{\mathrm{fake}}\right)∥}_{S}\leq \epsilon ,$$

where ϵ denotes a small semantic-space distance.

We refer to this limitation as a visual perception bottleneck in this paper. Due to this bottleneck, the language model L receives visual tokens with limited separability between authentic and forged inputs. Consequently, applying adaptation only on the language side can leave the model less sensitive to fine-grained forgery cues, thereby limiting cross-dataset generalization.

### 3.3. Inconsistency-Aware VLM Tuning (IncoTune)

To alleviate the perception bottleneck without full-parameter fine-tuning, we propose the IncoTune framework. Unlike the common setting that restricts parameter-efficient tuning mainly to the language side, IncoTune introduces trainable adapter updates into the visual encoder while keeping the original pre-trained weights frozen.

**Figure 2:**
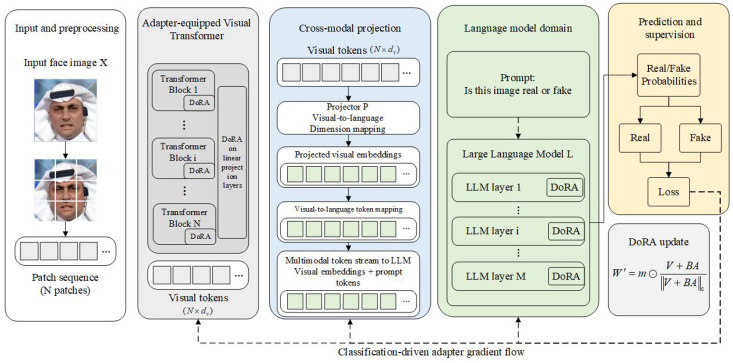
Overall architecture of the proposed IncoTune framework. Given an input image, the vision-adapted Vision Transformer extracts visual tokens, which are mapped into the LLM embedding space through a cross-modal projector. The original pre-trained weights are kept frozen, while DoRA modules are inserted into selected linear projection layers in both the visual encoder and the language model. The A/B token-level supervision provides a classification-oriented gradient flow that updates the trainable adapter parameters and adapts the visual token extraction process to task-relevant forgery cues.

The overall architecture of IncoTune is depicted in Figure 2. The cross-modal projector P maps visual tokens into the language embedding space. In our implementation, P is not fully fine-tuned as an independent module. Across the ablation variants evaluated in the ablation study, the training status of P is kept consistent. Specifically, we inject parameter-efficient adapters into the linear projection layers of the selected modules, including the Query, Key, Value, and MLP projections in the visual encoder V and the language model L. Let $W_{0}\in R^{d_{\mathrm{out}}\times d_{\mathrm{in}}}$ denote a pre-trained frozen weight matrix. The adapter-based forward pass is written as

(4) $$h=W_{0}x+\Delta Wx,$$

where ΔW is the trainable adaptation weight. By making ΔW trainable within the visual encoder, the gradients from the supervised A/B token prediction objective can update the vision-side adapter parameters. This adapter-based gradient flow encourages the vision-side adaptation modules to adjust the visual token extraction process, making the learned representation more sensitive to localized forgery cues.

### 3.4. Directional Low-Rank Adaptation

Standard Low-Rank Adaptation (LoRA) represents the weight update with a low-rank decomposition:

(5) ΔW=BA,

where $B\in R^{d_{\mathrm{out}}\times r}$, $A\in R^{r\times d_{\mathrm{in}}}$, and *r* is the rank. This design provides a parameter-efficient way to adapt the model, but the magnitude and direction of the update are coupled.

IncoTune employs Directional Low-Rank Adaptation (DoRA) [29]. DoRA represents the adapted weight using a magnitude vector $m\in R^{d_{\mathrm{out}}}$ and a directional matrix $V\in R^{d_{\mathrm{out}}\times d_{\mathrm{in}}}$:

(6) $$W^{\prime }=m⊙\frac{V+BA}{{∥V+BA∥}_{c}}.$$

where ⊙ denotes element-wise multiplication, and ${∥\cdot ∥}_{c}$ represents vector-wise normalization.

Within the IncoTune framework, the low-rank update BA provides trainable adaptation, while the magnitude vector *m* controls the scale of the adapted weights. This decomposition allows the model to adapt vision-side and LLM-side projections with a limited number of trainable parameters.

## 4. Experiments

### 4.1. Implementation Details

We adopt a unified preprocessing and evaluation pipeline for all model variants. For each input frame, we use the dlib library [30] to detect facial landmarks and perform face alignment and bounding-box cropping. Frames for which no valid face can be detected are excluded from the processed frame lists. The cropped facial regions are resized to 224×224 pixels before being fed into the visual encoder. The same preprocessing procedure is used for training, validation, and testing.

Unless otherwise specified, IncoTune uses Qwen2.5-VL-7B-Instruct as the foundational multimodal backbone. We adopt low-rank adapter tuning instead of full-parameter fine-tuning. For both DoRA and LoRA, the intrinsic rank *r* is set to 8, and the scaling factor α is set to 32. The maximum sequence length for the visual–textual input is set to 2048 tokens.

For binary decision making, we formulate frame-level deepfake detection as a two-choice visual question answering task. The textual prompt is: “<image> Is this a real image or a generated image? Just answer with A or B. A: real or B: generated.” The target answer is a single token, where A denotes a real frame and B denotes a fake frame. No additional classification head is introduced. During training, the model is optimized using the standard supervised fine-tuning objective on the target answer token. During inference, we read the log-probabilities of the first generated token corresponding to A and B, denoted as $\ell_{A}$ and $\ell_{B}$, and compute the normalized fake probability as

$$p_{B}=\frac{\exp(\ell_{B})}{\exp\left(\ell_{A}\right)+\exp\left(\ell_{B}\right)}.$$

The value of $p_{B}$ is used as the frame-level fake score for evaluation.

All experiments are implemented using PyTorch version 2.8.0+cu128 and the ms-swift version 3.12.3 training library. The model weights and optimizer states are cast to bfloat16 precision. We use the AdamW optimizer with an initial learning rate of $1\times 10^{-4}$, a weight decay of 0.01, and a warm-up ratio of 0.05. The model is trained for one pass over the training frame list. The same training schedule is used for IncoTune and all ablation variants. Training is conducted on 4× NVIDIA RTX 4090 GPUs. The per-device training batch size is 16, the per-device evaluation batch size is 16, and the gradient accumulation step is 4. The dataloader uses 20 workers.

The datasets used in our experiments include FaceForensics++ (FF++) [31], Celeb-DF-v2 (denoted as Celeb-DF in the following tables) [32], DFDC [33], DFDCP, and DFD. The sampled test lists are kept fixed for all model variants to ensure that the ablation comparison is conducted under the same evaluation protocol. The main cross-dataset comparison is reported using frame-level Area Under the Receiver Operating Characteristic Curve (AUC) [34], while complementary threshold-dependent metrics are reported for operating-point analysis.

### 4.2. Dataset Composition and Evaluation Protocol

Table 1 summarizes the frame-level dataset composition and the experimental frame lists used for model training, validation, and cross-dataset evaluation. All frames are processed after face detection, alignment, and frame filtering. FF++ is used to construct the training and validation lists, while Celeb-DF, DFD, DFDC, and DFDCP are used as target datasets. For each external target dataset, a fixed 10,000-frame evaluation list is sampled from the corresponding processed frame set. The same evaluation lists are used for all model variants to ensure consistent comparison across the ablation settings. All reported statistics are frame-level statistics after face processing, and the external evaluation lists are generated once after face filtering and kept unchanged across all model variants.

### 4.3. Cross-Dataset Generalization Results

Cross-dataset generalization is a major challenge in deepfake detection. Following the commonly used evaluation setting in face forgery detection, we train our model on an FF++-based training frame list and evaluate it on external datasets, including Celeb-DF, DFD, DFDC, and DFDCP. Since video-level methods may use temporal information or prediction aggregation across multiple frames, Table 2 focuses on representative frame-level methods.

As shown in Table 2, IncoTune obtains 91.7% AUC on Celeb-DF, 98.6% AUC on DFD, 81.3% AUC on DFDC, and 90.5% AUC on DFDCP. These results show that the proposed vision-side adaptation strategy improves frame-level cross-dataset detection across multiple unseen datasets under the evaluated setting.

The results on DFDC and DFDCP further show the difficulty of cross-dataset detection under more diverse real-world conditions. DFDC contains diverse generation sources and perturbations, where IncoTune obtains 81.3% AUC. On DFDCP, IncoTune reaches 90.5% AUC. These results indicate that IncoTune is effective under the listed frame-level protocol, while highly diverse datasets still remain challenging.

We further evaluate source-to-target transfer by using DFDCP as the source training dataset. The trained IncoTune model is then evaluated on FF++ and Celeb-DF. This setting complements the FF++-trained evaluation by changing the source dataset rather than only changing the target dataset.

As shown in Table 3, IncoTune achieves 83.84% AUC when trained on DFDCP and evaluated on FF++, suggesting that some forgery-related cues learned from DFDCP can transfer to FF++. The AUC on Celeb-DF is 76.95%, showing that transfer to Celeb-DF is more difficult under the DFDCP-source setting. These results suggest that the source dataset has a noticeable influence on cross-dataset performance.

The results of previous methods are cited from their original papers or benchmark reports. For the ablation studies, all variants are trained and evaluated using the same frame lists and inference pipeline.

### 4.4. Ablation Study on Adapter Placement and Type

To evaluate the effects of adapter placement and adapter type, we conduct an ablation study using LoRA and DoRA under frozen-vision and vision-adapted settings. Table 4 reports AUC values with 95% bootstrap confidence intervals computed on the fixed frame-level evaluation lists.

The first two rows compare LoRA and DoRA when the visual encoder is frozen. Language-side LoRA obtains an average AUC of 79.82%, while Language-side DoRA obtains 78.77%. This result indicates that replacing LoRA with DoRA alone does not improve cross-dataset generalization when the visual representation remains fixed.

The third row introduces LoRA adapters into both the visual and language pathways. Compared with Language-side LoRA, Vision+Language LoRA improves the average AUC from 79.82% to 89.51%. This result suggests that introducing trainable adaptation into the visual pathway is the main source of improvement in our setting.

The fourth row further replaces LoRA with DoRA in both pathways. IncoTune achieves the highest average AUC among the ablation variants, reaching 90.50%. Compared with Vision+Language LoRA, IncoTune improves the average AUC by 0.99 percentage points. This suggests that DoRA provides additional gains when combined with vision-side adaptation, while the larger improvement comes from adapting the visual pathway itself.

### 4.5. Statistical Significance and Multi-Seed Stability Analysis

To further examine the difference between Vision+Language LoRA and IncoTune, we perform paired bootstrap analysis on the same evaluation frame lists using 10,000 bootstrap resamples. The AUC difference is computed as IncoTune minus Vision+Language LoRA.

As shown in Table 5, IncoTune improves over Vision+Language LoRA on the evaluated datasets under paired bootstrap analysis. This result supports the additional effect of DoRA when the visual pathway is also adapted.

To assess the stability of IncoTune under different random initializations, we conduct a multi-seed analysis on the full FF++ training set. Specifically, IncoTune is trained using three random seeds and evaluated on Celeb-DF and DFDC, covering a high-quality external benchmark and a more diverse real-world benchmark.

As shown in Table 6, IncoTune shows stable performance across three independent training runs. The mean AUC reaches 91.40% on Celeb-DF and 81.74% on DFDC, which is consistent with the single-run results reported in Table 4. The standard deviations are 0.83 on Celeb-DF and 0.59 on DFDC, indicating that the reported performance is not highly sensitive to random initialization.

### 4.6. Threshold-Dependent Evaluation Metrics

We report threshold-related metrics for IncoTune to characterize its prediction behavior under fixed decision thresholds. Accuracy, F1-score, EER, APCER/BPCER, and sensitivity/specificity are reported under thresholds of 0.5, 0.6, and 0.7. The sensitivity/specificity and APCER/BPCER values are computed from the corresponding binary confusion matrices at each threshold.

As shown in Table 7, the complementary metrics vary with the decision threshold. Increasing the threshold generally improves specificity and reduces BPCER, while sensitivity decreases and APCER increases. This pattern reflects the expected trade-off between detecting forged frames and reducing false positives for real frames. On DFD, the model remains stable across the tested thresholds. On DFDC and DFDCP, the changes in sensitivity and specificity are more pronounced, indicating that the operating threshold has a stronger influence under more challenging cross-dataset conditions. These metrics provide threshold-specific evidence that complements the threshold-independent AUC results used in the main cross-dataset comparison.

### 4.7. Zero-Shot Baseline Analysis

To evaluate whether the detection ability comes directly from the original VLM or from task-specific adaptation, we further test the untuned Qwen2.5-VL-7B model under the same A/B decision format.

Table 8 shows that the untuned Qwen2.5-VL-7B model performs close to random guessing on several datasets under the same binary decision format. This indicates that task-specific adapter tuning is necessary for learning forgery-related decision cues.

### 4.8. Robustness Analysis

Deepfake images may undergo various post-processing operations during transmission and platform re-encoding. To evaluate the robustness of IncoTune under common image transformations, we test the trained model on Celeb-DF with resizing, center cropping, brightness adjustment, low-light transformation, contrast adjustment, saturation adjustment, and JPEG compression. All perturbations are applied after face cropping and resizing, and the same inference protocol is used as in the clean evaluation. The three severity levels are defined using fixed transformation parameters. Specifically, Level 1/2/3 correspond to resize scales of 0.75/0.50/0.25, center-crop ratios of 0.90/0.80/0.70, brightness offsets of +10/+20/+30, low-light factors of 0.85/0.70/0.55, contrast factors of 1.15/1.30/1.50, saturation factors of 1.20/1.50/1.80, and JPEG quality values of 75/50/25, respectively.

As shown in Table 9, IncoTune remains relatively stable under moderate resizing, cropping, brightness, low-light, contrast, and saturation changes. Strong resizing and JPEG compression lead to larger performance drops. In particular, the AUC under JPEG compression decreases from 91.66% under clean conditions to 70.15% at Level 3, indicating that severe compression remains a challenging post-processing condition.

### 4.9. t-SNE Visualization of Latent Features

To provide an auxiliary visualization of the latent feature distributions learned by different tuning strategies, we extract hidden features from the language-model normalization layer and project them into a two-dimensional space using t-SNE. For both models, we use the same Celeb-DF subset with 2000 frames, including 1000 real and 1000 fake frames. The t-SNE settings are fixed as follows: PCA initialization, perplexity 30, random seed 42, and automatic learning rate.

**Figure 3:**
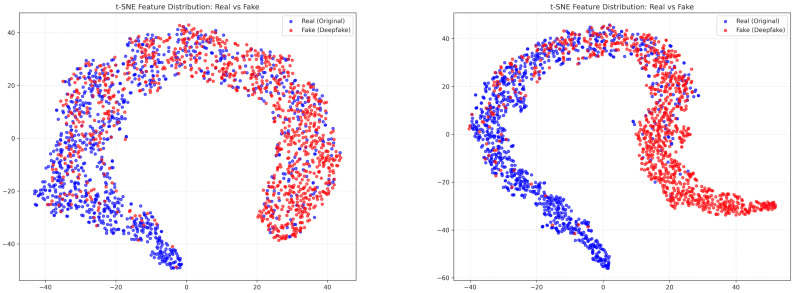
t-SNE visualization of latent features on Celeb-DF. The left plot corresponds to the frozen-vision LoRA baseline, and the right plot corresponds to the proposed IncoTune framework.

As shown in Figure 3, the frozen-vision LoRA baseline presents a more mixed visual distribution of real and fake samples, whereas IncoTune shows a more separated pattern on the selected Celeb-DF subset. Since t-SNE can be affected by sampling and hyperparameter settings, we use this figure only as a qualitative illustration. The main evidence for the effectiveness of vision-side adaptation is provided by the quantitative results in Table 4 and Table 5.

### 4.10. Model Capacity and Inference Cost Analysis

To provide a trainable-parameter reference, we compare the reported parameter scale and Celeb-DF AUC with several representative methods. As shown in Table 10, IncoTune updates 27.0M adapter parameters during training and achieves 91.66% AUC on Celeb-DF. This comparison is intended to summarize the trainable-parameter scale rather than deployment cost, since different methods use different backbones and training protocols. The inference latency and memory consumption of IncoTune are reported separately in Table 11.

Table 11 further reports the inference cost of Vision+Language LoRA and IncoTune. Since both variants use the same Qwen2.5-VL-7B backbone, their inference latency and peak GPU memory are similar. IncoTune is parameter-efficient in terms of trainable parameters during adaptation, but inference still requires a forward pass through the full VLM backbone.

## 5. Limitations

This study focuses on frame-level deepfake detection. The current framework does not explicitly model temporal consistency across consecutive frames, and therefore does not use motion-level cues that may be useful for video-level detection. In addition, although the robustness experiment evaluates several common image transformations, strong JPEG compression and severe resizing still cause noticeable performance drops. This suggests that repeated video compression and social-media transmission remain challenging scenarios. The current experiments are conducted using Qwen2.5-VL-7B-Instruct as the base VLM. Although the proposed adapter-placement strategy is conceptually not restricted to this backbone, its effectiveness on other VLM families, such as LLaVA and InternVL, has not been fully evaluated in this study.

IncoTune updates only adapter parameters during training, but inference still requires a forward pass through a large VLM backbone. Therefore, practical deployment should consider GPU memory consumption and inference latency. Future work will investigate temporal modeling, model compression, and more efficient inference strategies.

## 6. Conclusions

In this paper, we investigated the frozen-vision setting in VLM-based deepfake detection and analyzed its potential limitation for frame-level deepfake detection. A fixed visual encoder can preserve useful pre-trained representations, but it may be less adaptive to subtle local artifacts in deepfake images. To address this issue, we proposed IncoTune, an inconsistency-aware tuning framework that introduces trainable vision-side adaptation into the visual encoder and applies Directional Low-Rank Adaptation (DoRA) to selected linear projection layers in both the visual encoder and the language model. The original pre-trained weights are kept frozen, while the adapter parameters are trained to adapt the visual token extraction process to the deepfake detection task.

The experimental results show that IncoTune improves over the frozen-vision ablation baseline and achieves competitive frame-level generalization among representative frame-level detectors. The ablation study further shows that adapter placement has an important influence on cross-dataset performance. In our setting, replacing LoRA with DoRA under a frozen visual encoder does not improve the average AUC, whereas introducing trainable adaptation into the visual pathway produces a larger improvement. When DoRA is applied to both the visual encoder and the language model, IncoTune achieves the best average AUC among the ablation variants. The paired bootstrap analysis and multi-seed stability analysis further support the reliability of the observed improvement.

The zero-shot, robustness, visualization, and inference-cost analyses provide complementary evidence about the behavior of IncoTune. The zero-shot experiment shows that the untuned Qwen2.5-VL model performs close to random guessing on several datasets, indicating that task-specific adapter tuning is necessary for deepfake detection. The extended robustness experiment reports the behavior of IncoTune under resizing, cropping, brightness, low-light, contrast, saturation, and JPEG compression. The results show that the model remains relatively stable under moderate transformations, while strong JPEG compression remains challenging. The t-SNE visualization provides a qualitative view of the learned latent space, and the cost analysis shows that IncoTune introduces no obvious inference overhead compared with Vision+Language LoRA under the same VLM backbone.

Future work will explore temporal modeling, cross-backbone validation, broader real-world robustness evaluation, and more efficient deployment strategies for VLM-based deepfake detection.

## Figures and Tables

**Table 1:** Dataset composition and experimental frame lists used for model training, validation, and cross-dataset evaluation.

Dataset	Experimental Role	Processed Frame Set			Experimental Frame List		
		Total	Real	Fake	Total	Real	Fake
FF++	Training	504,564	91,250	413,314	504,564	91,250	413,314
FF++	Validation	10,000	1848	8152	10,000	1848	8152
Celeb-DF	Testing	198,427	18,650	179,777	10,000	1301	8699
DFD	Testing	201,980	18,979	183,001	10,000	1000	9000
DFDC	Testing	126,941	60,859	66,082	10,000	4703	5297
DFDCP	Testing	80,297	19,899	60,398	10,000	2628	7372

**Table 2:** Representative frame-level cross-dataset results on deepfake datasets. The results are reported in terms of frame-level AUC (%). Results of prior methods are cited from their original papers or benchmark reports.

Model	Pub.	Celeb-DF	DFD	DFDC	DFDCP
F3Net [6]	ECCV’20	73.5	79.8	70.2	73.5
Face X-Ray [4]	CVPR’20	80.6	95.4	80.9	-
DCL [16]	AAAI’22	82.3	91.6	76.7	-
RECCE [12]	CVPR’22	82.3	89.1	69.6	73.4
UCF [17]	ECCV’23	82.4	94.5	80.5	-
CFM [9]	TIFS’24	89.7	95.2	70.6	80.2
LSDA [13]	CVPR’24	83.0	88.0	73.6	81.5
ProDet [35]	NeurIPS’24	90.0	-	72.4	81.1
UDD [15]	AAAI’25	86.9	91.0	75.8	85.6
ForAda [24]	CVPR’25	90.0	93.3	**84.3**	89.0
RepDFD [23]	AAAI’25	80.0	-	77.3	**90.6**
**IncoTune (Ours)**	-	**91.7**	**98.6**	81.3	90.5

Note: “-” indicates that the corresponding result was not reported in the cited source; “-” in the Pub. column denotes the proposed method. Bold values indicate the best performance in each dataset column; the bold model name denotes the proposed method.

**Table 3:** Cross-source generalization results of IncoTune under DFDCP-source training. The model is trained on DFDCP and evaluated on FF++ and Celeb-DF. AUC (%) is reported.

Training Dataset	Test Dataset	AUC
DFDCP	FF++	83.84
DFDCP	Celeb-DF	76.95

**Table 4:** Ablation results with 95% bootstrap confidence intervals. AUC (%) is reported for each test dataset.

Method	Visual Encoder	Adapter	Celeb-DF	DFD	DFDC	DFDCP	Avg.
Language-side LoRA	Frozen	LoRA	77.72 (76.59–78.85)	92.97 (92.42–93.52)	72.77 (71.79–73.75)	75.82 (74.85–76.79)	79.82
Language-side DoRA	Frozen	DoRA	75.87 (74.41–77.29)	92.52 (91.95–93.09)	72.01 (71.02–73.02)	74.66 (73.56–75.71)	78.77
Vision + Language LoRA	Adapted	LoRA	90.01 (89.35–90.67)	97.97 (97.73–98.21)	80.67 (79.83–81.51)	89.39 (88.78–90.00)	89.51
**IncoTune**	Adapted	DoRA	**91.66** **(90.88–92.34)**	**98.57** **(98.38–98.76)**	**81.27** **(80.44–82.10)**	**90.49** **(89.92–91.06)**	**90.50**

Note: Bold values indicate the best performance among the ablation variants.

**Table 5:** Paired bootstrap comparison between Vision+Language LoRA and IncoTune. The difference is computed as IncoTune minus Vision+Language LoRA in AUC percentage points.

Dataset	VL-LoRA AUC	IncoTune AUC	Difference	95% CI of Difference	*p*-Value
Celeb-DF	90.01	91.66	+1.65	[+1.26, +2.04]	<0.001
DFD	97.97	98.57	+0.60	[+0.34, +0.86]	<0.001
DFDC	80.67	81.27	+0.60	[+0.32, +0.88]	<0.001
DFDCP	89.39	90.49	+1.10	[+0.81, +1.39]	<0.001

**Table 6:** Multi-seed stability analysis of IncoTune on the full FF++ training set. The model is trained with three random seeds and evaluated on Celeb-DF and DFDC. AUC (%) is reported for each seed, and the mean and standard deviation are computed over three seeds.

Training Set	Model	Test Dataset	Seed 42	Seed 123	Seed 2026	Mean ± Std
FF++	IncoTune	Celeb-DF	90.63	91.31	92.28	91.40 ± 0.83
FF++	IncoTune	DFDC	81.32	81.49	82.41	81.74 ± 0.59

**Table 7:** Complementary evaluation metrics of IncoTune under different fixed decision thresholds. All values except the threshold are reported in percentage. EER is included as a threshold-independent reference, while the other metrics are computed at each fixed threshold.

Dataset	Threshold	Acc.	F1	EER	APCER	BPCER	Sens.	Spec.
Celeb-DF	0.5	90.64	94.80	16.10	1.90	59.26	98.10	40.74
Celeb-DF	0.6	90.65	94.78	16.10	2.43	55.65	97.57	44.35
Celeb-DF	0.7	90.73	94.80	16.10	2.92	51.73	97.08	48.27
DFD	0.5	98.28	99.05	4.98	0.91	9.00	99.09	91.00
DFD	0.6	98.31	99.06	4.98	1.08	7.20	98.92	92.80
DFD	0.7	98.25	99.02	4.98	1.30	5.80	98.70	94.20
DFDC	0.5	72.06	75.35	26.62	19.39	37.57	80.61	62.43
DFDC	0.6	72.38	74.98	26.62	21.88	34.08	78.12	65.92
DFDC	0.7	72.95	75.06	26.62	23.16	31.43	76.84	68.57
DFDCP	0.5	84.19	89.31	18.48	10.38	31.05	89.62	68.95
DFDCP	0.6	83.85	88.94	18.48	11.90	28.08	88.10	71.92
DFDCP	0.7	83.43	88.53	18.48	13.28	25.80	86.72	74.20

**Table 8:** Zero-shot and tuned Qwen2.5-VL comparison. AUC (%) is reported for each dataset.

Method	Tuning	Celeb-DF	DFD	DFDC	DFDCP
Qwen2.5-VL-7B	None	52.65	58.97	50.35	54.07
IncoTune	Adapter tuning	91.66	98.57	81.27	90.49
Gain	-	+39.01	+39.60	+30.92	+36.42

Note: “-” denotes not applicable.

**Table 9:** Extended robustness evaluation of IncoTune on Celeb-DF. AUC (%) is reported under different perturbation types and severity levels.

Perturbation	Clean/L0	Level 1	Level 2	Level 3
Clean	91.66	-	-	-
Resize	-	91.76	90.19	84.22
Center crop	-	90.64	89.01	87.00
Brightness	-	91.26	90.80	90.11
Low light	-	91.54	91.28	90.09
Contrast	-	90.98	89.83	88.10
Saturation	-	91.53	90.99	90.20
JPEG	-	89.19	82.79	70.15

Note: “-” denotes not applicable.

**Table 10:** Trainable-parameter reference and Celeb-DF performance comparison. AUC (%) is reported. For VLM-based methods, the parameter number refers to trainable adapter parameters.

Model	Reported Params	Celeb-DF
RepDFD [23]	0.078 M	80.0
DCL [16]	19.35 M	82.3
RECCE [12]	25.83 M	82.3
SBI [5]	19.34 M	88.6
ViT-B [36]	85.8 M	79.4
CFM [9]	15.51 M	89.7
EVL [37]	53.8 M	80.3
**IncoTune (Ours)**	**27.0 M trainable**	**91.7**

Note: Bold indicates the proposed method and its corresponding result.

**Table 11:** Inference cost comparison on Celeb-DF. Latency is measured in seconds per frame on 300 frames after 20 warm-up samples using a single RTX 4090 GPU.

Method	Samples	Mean	Median	P95	FPS	Mem.
VL-LoRA	300	0.1343	0.1306	0.1585	7.45	15.48 GB
IncoTune	300	0.1341	0.1309	0.1591	7.46	15.48 GB

## Data Availability

The public datasets used in this study are available from their official sources under their respective access policies. The processed frame lists, evaluation split files, textual prompts, main hyperparameter configurations, and evaluation scripts are available from the corresponding author upon reasonable request. The full training code and trained model weights are not publicly released at this stage, but may be made available for non-commercial research verification upon reasonable request and subject to approval.
